# Bridging Clinical Phenotypes and Histopathological Endotypes in Refractory Chronic Cough

**DOI:** 10.3390/jcm15176579

**Published:** 2026-08-26

**Authors:** Karolina Klimowicz, Katarzyna Białek-Gosk, Elżbieta Magdalena Grabczak, Olga Truba, Joanna Żuchowska, Agata Cyran, Magdalena Paplińska-Goryca, Patrycja Nejman-Gryz, Marta Dąbrowska

**Affiliations:** 1 Department of Internal Medicine, Pulmonary Diseases and Allergy, Medical University of Warsaw, 02-091 Warsaw, Poland; karolinaakrakowiak@gmail.com (K.K.); katarzyna.bialek-gosk@wum.edu.pl (K.B.-G.); elzbieta.grabczak@wum.edu.pl (E.M.G.); olga.truba@wum.edu.pl (O.T.); magdalena.paplinska@wum.edu.pl (M.P.-G.); patrycja.nejman-gryz@wum.edu.pl (P.N.-G.); 2 2nd Department of Clinical Radiology, Medical University of Warsaw, 02-091 Warsaw, Poland; jzuchowska1@gmail.com; 3 Department of Histopathology, Medical University of Warsaw, 02-091 Warsaw, Poland; aacyran@gmail.com

**Keywords:** refractory chronic cough, clinical phenotypes, histopathological endotypes, airway remodelling, bronchoalveolar lavage

## Abstract

**Background:** Refractory chronic cough (RCC) is a heterogeneous condition in which clinical phenotyping is used to guide treatment, yet its relationship to the underlying airway pathology remains poorly defined. This study assessed the concordance between clinically defined cough phenotypes and histopathological endotypes derived from bronchial biopsy and bronchoalveolar lavage fluid (BALF) in patients with RCC and compared chest computed tomography (CT) findings across endotypes. **Methods:** A prospective cross-sectional study was conducted in the cough centre between 2021 and 2025. Adults with RCC lasting more than six months and unresponsive to at least two pharmacological attempts underwent clinical phenotyping (eosinophilic, chronic bronchitis-related, or other) based on clinical symptoms, comorbidities, chest CT, spirometry, FeNO and blood eosinophil count with the assumption that clinical phenotypes might overlap. Histopathological endotype (eosinophilic, neutrophilic, mixed inflammatory, or normal) was assigned based on histopathological assessment of bronchial biopsy and BALF analysis. **Results:** A total of 30 patients were enrolled [22 women, 73.3%; median age 54 years (IQR 43–63) median cough duration 36 months (IQR 12–72)]. Clinically, an eosinophilic phenotype was identified in 11 patients, a chronic bronchitis phenotype in 15, and other phenotypes in 18, with frequent overlap. Airway inflammation or remodelling were present on histopathology in 27 of 30 patients (90%); a mixed inflammatory endotype predominated (15 patients), followed by eosinophilic (10), normal (3), and neutrophilic (2). Concordance between clinical phenotype and histopathological endotype was observed in 21 of 30 patients (70%). Subtle CT features of small-airway inflammation were found in 61.3% of patients but did not differ across endotypes. **Conclusions:** These findings demonstrate limited agreement between clinical phenotypes and tissue-level pathology in RCC, indicating that clinical phenotyping alone may be insufficient to capture its cellular and structural heterogeneity.

## 1. Introduction

Chronic cough (lasting over 8 weeks) affects up to 10% of adults worldwide and remains both a diagnostic and therapeutic challenge [1]. It may result from numerous conditions, including respiratory diseases as well as disorders originating outside the respiratory system. Reducing exposure of the airways to irritant factors, such as cigarette smoke or occupational exposures, and discontinuing medications that may induce cough constitute the first step in the management of chronic cough [2].

Causal treatment of diseases that induce cough (such as chronic pulmonary infections, COPD, asthma, interstitial lung diseases, chronic rhinitis or rhinosinusitis, and gastroesophageal reflux disease) leads to improvement or resolution of cough in many patients [3].

However, in a subset of individuals, chronic cough persists despite discontinuation of irritants and optimal management of underlying conditions, leading to the diagnosis of refractory chronic cough (RCC). RCC is typically burdensome, intense, long-lasting, and significantly impairs patients’ quality of life [4,5]. The key mechanism underlying RCC lies in heightened cough-reflex sensitivity to various low-intensity stimuli, such as changes in ambient temperature, strong odours, or aerosols (hypertussia), and even to non-irritant activities like talking, laughing, or deep breathing (allotussia). This results from sensitization of cough receptors within peripheral vagal nerve fibres, as well as central sensitization of the cough-reflex arc. Laryngeal paraesthesia, manifesting as an increased urge to cough, often coexists with these abnormalities [6]. Management of RCC is challenging and requires both pharmacological and non-pharmacological treatment approaches [2,7,8].

Importantly, a long-lasting cough, through mechanical, repetitive injury and multiple abrupt changes in intrathoracic pressure, may itself lead to damage of the airway epithelium, airway inflammation, and consequently airway remodelling [9].

Chronic cough can be classified in several ways, depending on the clinical or research perspective. The most traditional approach is based on aetiology and treatable traits, such as smoking or other chronic airway exposure to irritants, asthma, eosinophilic bronchitis, gastroesophageal reflux, upper airway diseases, laryngeal dysfunction, obesity-related traits, chronic infection, or presence of cough hypersensitivity [2,3,6]. More recently, data-driven clinical phenotypes derived from cluster analyses have been proposed to better capture heterogeneity in symptom patterns and disease severity [10].

Certain treatable traits can be relatively easily identified in routine practice; for example, eosinophilic airway inflammation suggested by elevated blood eosinophil count (BEC) or fractional exhaled nitric oxide (FeNO) may indicate a higher likelihood of response to inhaled corticosteroids [11,12,13,14]. Analogously, the presence of productive cough suggests chronic bronchitis related to infection or COPD and indicates the need for lung function monitoring, infection diagnostics, airway clearance support (mucolytics and physiotherapy), or prolonged azithromycin therapy [7,15,16]. Thus, clinical identification of phenotypes such as eosinophilic inflammation or chronic bronchitis may guide treatment selection and improve therapeutic effectiveness. Despite these strategies, treatment efficacy in chronic cough remains limited, and RCC is frequently diagnosed.

To date, studies investigating inflammatory changes and airway remodelling associated with chronic cough have documented structural alterations such as subepithelial basement membrane thickening, epithelial desquamation, goblet cell hyperplasia, and an infiltration of inflammatory cells—including mast cells, eosinophils, and lymphocytes—as well as an increased density of intraepithelial sensory nerve fibres [17]. However, assessment of airway inflammation and remodelling require specialized histopathological evaluation, which are not routinely performed in everyday clinical practice. Current guidelines suggest diagnosing RCC based on clinical features—airway hypersensitivity symptoms and lack of treatment response—rather than histological confirmation. On the other hand, demonstrating airway remodelling features, simultaneously excluding eosinophilic inflammation or neutrophilic inflammation, together with clinical exclusion of other aetiology of treatable traits might provide guidance for further management of patients with RCC.

Although chest CT is not routinely recommended for all patients with chronic cough, recent studies indicate that beyond identifying bronchiectasis, interstitial abnormalities, or parenchymal lesions, CT may also reveal features of small-airway inflammation, such as airway wall thickening, mucus plugging, or ventilation defects, suggesting bronchiolar involvement [18]. Similarly, there is no role for routine bronchoscopy for most patients with CC, but it may occur useful in some situations such as suspicion of tracheal or main bronchi abnormalities including collapsibility or intrabronchial tumour or foreign body or in productive chronic cough [7] Recently, bronchoscopy proved useful in patients with productive RCC by identifying specific treatable traits, including neutrophilic airway inflammation [19].

Taking these considerations into account, we designed a study assessing the concordance between selected clinical phenotypes and histopathological findings from bronchial wall biopsies combined with bronchoalveolar lavage fluid (BALF) analysis in patients with RCC.

## 2. Methods

### 2.1. General Study Design

A prospective cross-sectional observational study was performed in the cough centre at the Department of Internal Medicine, Pulmonary Diseases and Allergy at Medical University of Warsaw between 2021 and 2025. The project has been approved by the Institutional Review Board of the Medical University of Warsaw (KB/134/2019) and retrospectively registered (12 June 2026) in the Clinical Trials Registry (NCT07659860). The delay in registering the study in ClinicalTrials.gov resulted from administrative disruptions related to the COVID-19 pandemic. This timing did not affect the study protocol, data collection, or patient safety.

The primary outcome of the analysis was the assessment of the concordance between the clinical phenotype of RCC and the presence of features or eosinophilic, neutrophilic and mixed inflammation, as well as remodelling identified in bronchial mucosal biopsy specimens and BALF. The secondary aim was to compare the presence of chest CT abnormalities related to airway inflammation across histopathological endotypes.

### 2.2. Patients

Patients with refractory chronic cough lasting more than 6 months who were referred with a diagnosis of cough as the main or isolated ailment in the Department of Internal Medicine, Pulmonary Diseases and Allergy of the Medical University of Warsaw were invited to participate in the study. All patients included provided written informed consent.

Patients were eligible for inclusion if they were 18–75 years old; had chronic cough lasting more than 6 months that was unresponsive to previous treatment (with at least two prior unsuccessful pharmacological attempts); presented with a normal or near-normal chest radiograph or only abnormalities considered irrelevant to cough aetiology; and had clinical indications for bronchoscopy, including productive cough, CT abnormalities such as mucus plugs or bronchiectasis, suspected tracheobronchomalacia, assessment of eosinophilic airway inflammation when induced sputum could not be obtained, or any suspicion or high risk of malignancy. Additional criteria included the absence of respiratory tract infection within the preceding 6 weeks and a negative smoking history (≥6 months of abstinence) and no history of ACE inhibitor use. During the study, patients were treated according to the diagnosed chronic cough aetiology.

Exclusion criteria included age < 18 or >75 years; cough duration shorter than 6 months; active smoking or abstinence shorter than 6 months; respiratory tract infection within 6 weeks prior to enrolment or occurring during the study (with the option of re-evaluation after another 6 weeks); an established diagnosis of lung malignancy, chronic lung infection or interstitial lung disease; and any contraindications to performing lung function testing or bronchoscopy.

### 2.3. Definitions and Study Design

Cough severity was assessed using a Visual Analogue Scale (VAS), with clinical improvement defined as a documented reduction in the VAS score. Additionally, the impact of cough on the patients’ health-related quality of life was evaluated using the validated Leicester Cough Questionnaire (LCQ).

Based on the identified treatable traits, prior diagnostic work-up, chest CT image analysis, spirometry with bronchodilator testing, FeNO, and blood eosinophil count (BEC), the clinical phenotype of each patient was assessed. The following clinical phenotypes were distinguished:

1. Clinical eosinophilic phenotype—assigned if eosinophilic asthma or eosinophilic bronchitis was diagnosed, or by evidence of eosinophilic airway inflammation, including FeNO > 25 ppb or BEC > 250 cells/µL [20,21].
2. Chronic bronchitis-related phenotype—assigned if the patient reported productive cough or if COPD was diagnosed or if chest CT demonstrated features consistent with bronchial inflammation (at least 3 out of 5: bronchial wall thickening, mucus plugs, bronchiectasis, peribronchial “tree-in-bud” opacities, heterogeneous air-trapping).
3. Other clinical phenotypes—including those related to gastroesophageal reflux (GER), upper airway cough syndrome (UACS), obesity, others or idiopathic cough.

Few clinical phenotypes could coexist within the same patient.

The criteria for diagnosing clinical phenotypes were based on BTS statement [7] excluding chest CT based criteria (at least 3 out of 5 features consistent with bronchial inflammation), which was arbitrary.

Algorithms for diagnosis asthma, eosinophilic bronchitis, GER, UACS as a CC reason were based on ERS recommendations and BTS statement [2,7]. The summary of diagnostic protocol is given in Supplementary Materials (Figure S1).

The pathomorphological endotype was diagnosed based on bronchial biopsy and analysis of BALF. Bronchoscopy was performed under local anaesthesia with lignocaine and conscious sedation using midazolam and fentanyl, with an Olympus video bronchoscope (BS-1TH190 Olympus Medical Systems Corp., Japan). During the procedure, airway anatomy was assessed, two mucosal forceps biopsies from the central bronchi were obtained for histopathological examination, and BALF was collected for cellular analysis and for both non-specific and specific cultures. BAL analysis was performed by two experienced researchers (PNG and MPG) blinded to the histopathological assessment. Histopathological assessment was performed by an experienced pathologist (AC) who was blinded to the patients’ clinical phenotypes. Based on histopathological assessment of bronchial biopsy and BALF analysis [22], the following patterns were identified:

1. Eosinophilic endotype—defined as an inflammatory infiltrate with eosinophil predominance in bronchial biopsies or more than 1% eosinophils in BALF.
2. Neutrophilic endotype—defined as an inflammatory infiltrate with neutrophil predominance in biopsies or more than 3% neutrophils in BALF.
3. Mixed inflammatory endotype—defined as a mixed infiltrate in biopsies, composed mainly of lymphocytes and neutrophils with a possible minor eosinophil component, and/or >15% lymphocytes and/or >3% neutrophils in BALF.
4. Normal findings—normal histology of bronchial biopsy and normal BALF cellular composition.

The concordance between the clinical phenotype and histopathological endotype was defined as follows:

1. Eosinophilic phenotype—when both the clinical assessment and biopsy findings or BALF indicated eosinophilic inflammation.
2. Chronic bronchitis-related phenotype—when the clinical phenotype corresponded to neutrophilic or a mixed inflammatory infiltrate on histopathology or BALF.
3. Other phenotypes—when the clinical phenotypes corresponded to a mixed inflammatory infiltrate on histopathology or BALF or normal histology and normal BALF cellular composition.

### 2.4. Computed Tomography (CT) of the Chest

Computed tomography (CT) of the chest was performed with a 16-row CT scanner (LightSpeed 16 General Electric Healthcare, Milwaukee, WI, USA) using 140 kV peak, 100–250 mA current and matrix size 512 × 512 and 1.00 or 1.25 mm collimation., without contrast injection. If regional aeration disorders were suspected, additional scans on the expiration were performed to assess presence of air trapping or mosaic attenuation. The CT scans were evaluated by an experienced chest radiologist (JZ) including evaluation of the presence, extent, and severity of bronchial wall thickening, bronchiectasis, and mucus plugs. Small airway pathology was identified by the presence of centrilobular abnormalities, including the tree-in-bud pattern. CT abnormality score was defined as sum of abnormalities identified in chest CT.

### 2.5. Spirometry and FENO

Baseline spirometry was performed using a calibrated spirometer the Vyntus (CareFusion, Germany) in adherence to the current American Thoracic Society and European Respiratory Society (ATS/ERS) standardization guidelines [23]. Absolute and percent predicted values for forced vital capacity (FVC) and forced expiratory volume in 1 s (FEV1) were derived using the Global Lung Function Initiative (GLI) reference equations.

Fractional exhaled nitric oxide (FeNO) was measured prior to any forced expiratory manoeuvres using the Medisoft Vyntus FeNO analyser (CareFusion, Germany) in accordance with the ATS/ERS clinical practice guidelines [24]. Patients performed a continuous exhalation at a constant flow rate of 50 mL/s, while ambient nitric oxide was dynamically excluded by the device’s internal scrubber. At least two reproducible exhalations were obtained for each subject, and the mean value was recorded in parts per billion (ppb).

### 2.6. Bronchoalveolar Lavage Fluid Analysis

Bronchoalveolar lavage was performed in the middle lobe or lingula by wedging the bronchoscope into the appropriate segmental bronchus. Three aliquots of 50 mL sterile saline solution (0.9% NaCl) prewarmed to 37 °C were instilled sequentially. BALF was subsequently aspirated using gentle suction and collected into sterile containers. Samples with low recovery volume were excluded from cytological analysis. Further BALF processing was performed in accordance with the recommendations of the American Thoracic Society [22]. Cytological evaluation was conducted in May–Grünwald–Giemsa-stained smears, and differential cell counts were determined by light microscopy. Additionally, BALF samples underwent standard microbiological analysis, including microscopic examination, aerobic and anaerobic cultures, and testing for the growth of acid-fast bacilli.

### 2.7. Assessment of Bronchial Biopsy

Bronchial biopsy specimens were fixed in formalin, embedded in paraffin, and processed according to standard histopathological protocols. Slides were stained with haematoxylin and eosin, with additional immunohistochemical markers applied when required to characterize inflammatory cell populations. Histopathological assessment focused on epithelial shedding, features of airway remodelling, and the intensity and composition of the inflammatory infiltrate within the biopsy specimens. Inflammation severity was assessed semi-quantitatively based on the density, composition, and distribution of inflammatory cells within the epithelial and subepithelial compartments. Mild inflammation was defined as a sparse infiltrate, moderate inflammation as a continuous and clearly visible infiltrate, and severe inflammation as a dense infiltrate occupying a substantial portion of the mucosal and submucosal compartments. All evaluations were performed by an experienced pulmonary pathologist blinded to clinical data.

### 2.8. Statistics

Descriptive data are presented as median and interquartile range or as number and percentage. Group comparisons were performed using non-parametric tests (Mann–Whitney U test, Kruskal–Wallis test, χ^2^ test or Fisher’s exact test, as appropriate). Concordance between clinical and histopathological phenotypes was assessed using accuracy, with concordance defined as the histopathological phenotype matching any of the clinical phenotypes assigned to a given patient. Due to the exploratory nature of the study, no formal sample size calculation was performed. Based on our previous studies [21], we assumed that the eosinophilic phenotype would be present in 40–50% of patients with chronic cough, whereas the neutrophilic phenotype would occur in approximately 20–25%.

Microsoft Copilot was used to support English language editing. All generated suggestions were reviewed, corrected when necessary, and fully approved by the authors, who retain complete responsibility for the final manuscript.

## 3. Results

### 3.1. Patients’ Characteristics

A total of 30 participants with RCC were enrolled in the study: 22 females (73.3%) and 8 males (26.7%), median age of patients was 54 years (IQR 43–63) and a median BMI 26.2 kg/m^2^ (IQR 23.3–30). Eleven participants were former smokers; nineteen had never smoked.

The median cough duration was 36 months (IQR 12–72; range 6–240 months). Nineteen participants reported dry cough, and eleven subjects had productive cough. In the whole cohort, the median cough severity on the Visual Analogue Scale (VAS) was 50 mm (IQR 32.5–73.5), and health-related quality of life assessed using the Leicester Cough Questionnaire (LCQ) was 10.55/21 points (IQR 8.78–13.03).

Asthma was diagnosed in 20 patients, gastroesophageal reflux in 17, and upper airway diseases in 19 patients. Some 10 participants had single cough-related diseases, while 20 patients had at least two coexisting cough-associated diseases. Before the study, patients were treated as follows:

- CC related to asthma: LABA + ICS (all 20 patients), with optional add-on LTRA (13 patients), and long-acting antimuscarinic agents (11 patients).
- CC related to GER: dietary modification (17 patients), proton pump inhibitors (PPI) (5 patients), and itopride (4 patients).
- UACS: intranasal corticosteroids (12 patients) and antihistamines (5 patients).

There were no differences in patients’ and cough characteristics between patients with asthma and other cough reasons (Table 1).

Among all patients, the median BEC was 165 cell/µL (IQR 70–230). In eight patients, BEC was equal or higher than 250 cells/µL. The median FeNO was 13.1 ppb (IQR 10.2–18), and only five patients had FeNO higher than 25 ppb. Spirometry was normal in 20 subjects, while 5 subjects presented irreversible airway obstruction, 4 presented reversible airway obstruction, and in 1 case, there were abnormalities suggestive for restrictive pattern.

**Figure 1:**
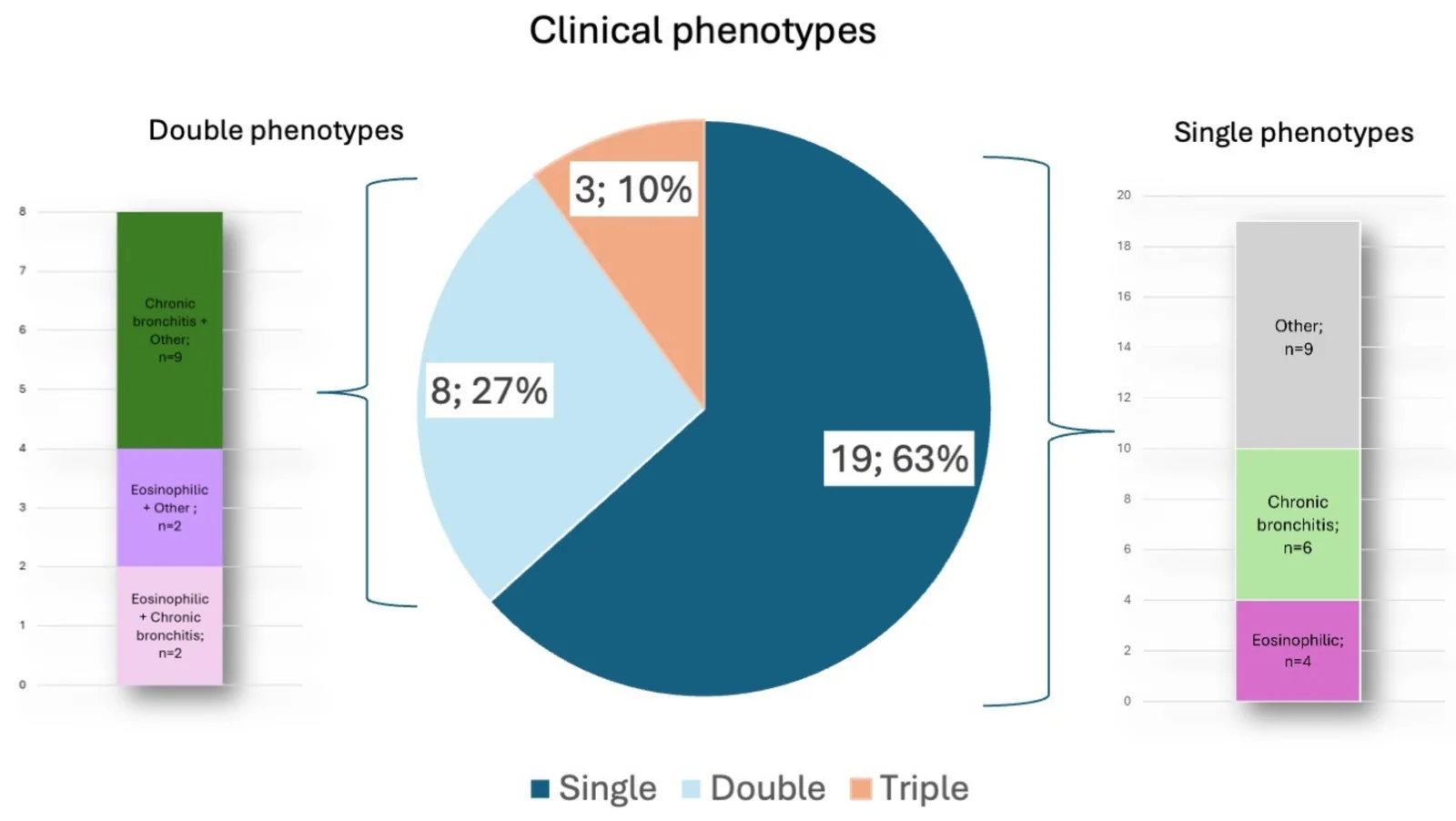
Distribution of clinical phenotypes.

Based on clinical evidence, an eosinophilic phenotype was identified in 11 patients (10 in the asthma group, 1 in the group without asthma), a chronic bronchitis phenotype in 15 patients (10 in the asthma group, 5 in the group without asthma), and other phenotypes in 18 patients (10 in the asthma group, 8 in the group without asthma) (Figure 1). As clinical phenotypes might have coexisted, the number of identified phenotypes exceeds the number of patients.

### 3.2. Bronchoscopy Findings

During bronchoscopy, swelling of tracheal and bronchial mucosa was found in 16/30 (51.6%). Next, an excessive dynamic airway collapse (EDAC) was identified in three patients, while marked bronchial malformations and distortions were present in two patients.

### 3.3. Histopathological Assessment of Bronchial Biopsy Specimens

**Figure 2:**
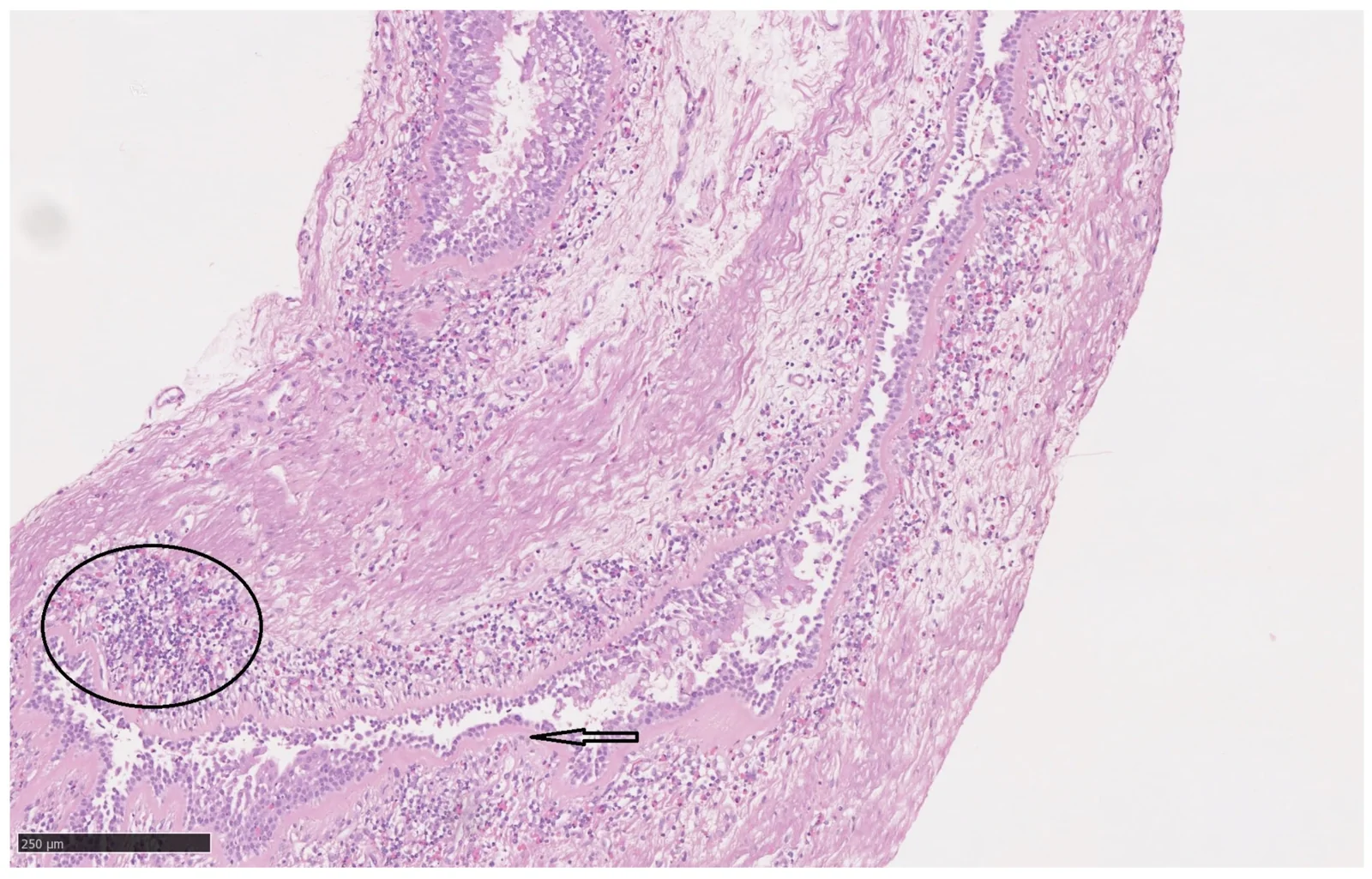
Inflammation and remodelling features in bronchial biopsy in patient with chronic cough. The circled area demonstrates a chronic inflammatory infiltrate with numerous eosinophils. The arrow indicates basement membrane thickening.

Airway inflammation or remodelling features were found in 27 of 30 patients (90%). Reticular basement membrane thickening was identified in 19 patients, oedema of submucosa in 5 subjects, infiltration of inflammatory cells in 21 patients (mild to moderate in 19 and severe in 3 specimens), and subbasement membrane (SBM) thickening in 15 patients (Figure 2).

Chronic inflammatory infiltrates within the bronchial mucosa were observed in sixteen cases (composed mainly of lymphocytes with occasional eosinophils); only one patient showed numerous eosinophils.

### 3.4. BALF Analysis

Analysis of bronchoalveolar lavage fluid revealed a normal BALF cellular profile in only four patients. In sixteen patients, more than 15% lymphocytes were identified in BALF; likewise, sixteen patients had neutrophilia >3%. BALF with an elevated eosinophil percentage >1% was found in twelve patients. Detailed cellular composition parameters are presented in Supplementary Table S1.

Microbiological analysis of BALF showed growth of *Haemophilus influenzae* (>10^4^ CFU/mL) in three patients, while *Mycobacterium kansasii* was isolated in one patient.

**Figure 3:**
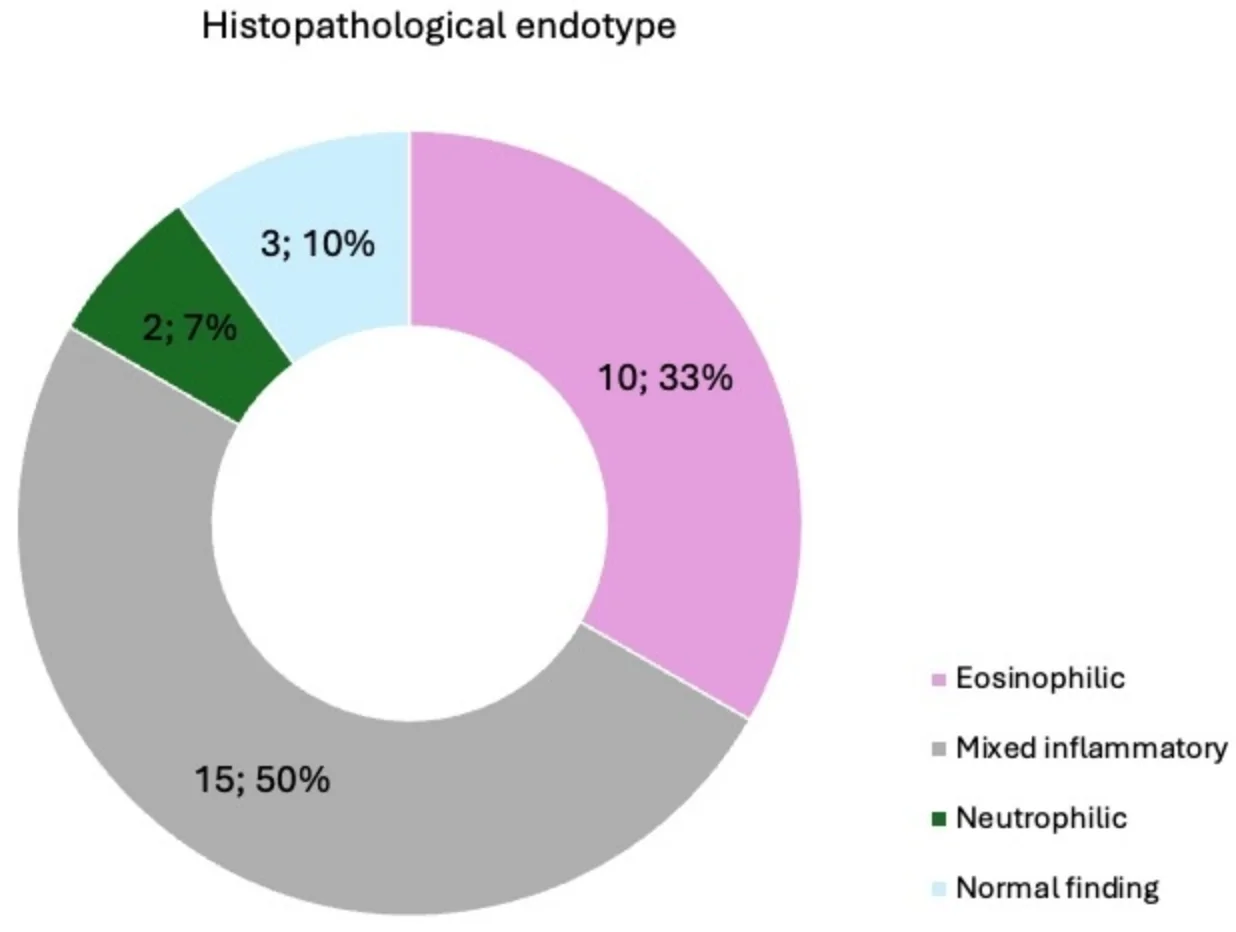
Distribution of histopathological endotypes.

Based on the histopathological assessment of bronchial biopsy specimens and BALF analysis, an eosinophilic endotype was identified in 10 patients, a mixed (lymphocytic and neutrophilic) inflammatory endotype in 15 patients, a neutrophilic endotype in 2 patients, and normal structure of bronchial wall in 3 patients (Figure 3). Histopathological endotypes were mutually exclusive, with only one endotype assigned to each patient.

### 3.5. Concordance Between Clinical and Histopathological Endotypes

In the analysis of all 30 cases, concordance between clinical phenotypes and histopathological endotypes was observed only in 21 of 30 patients (70%). Similar concordance was found in both eosinophilic and chronic bronchitis phenotypes (Table 2).

**Figure 4:**
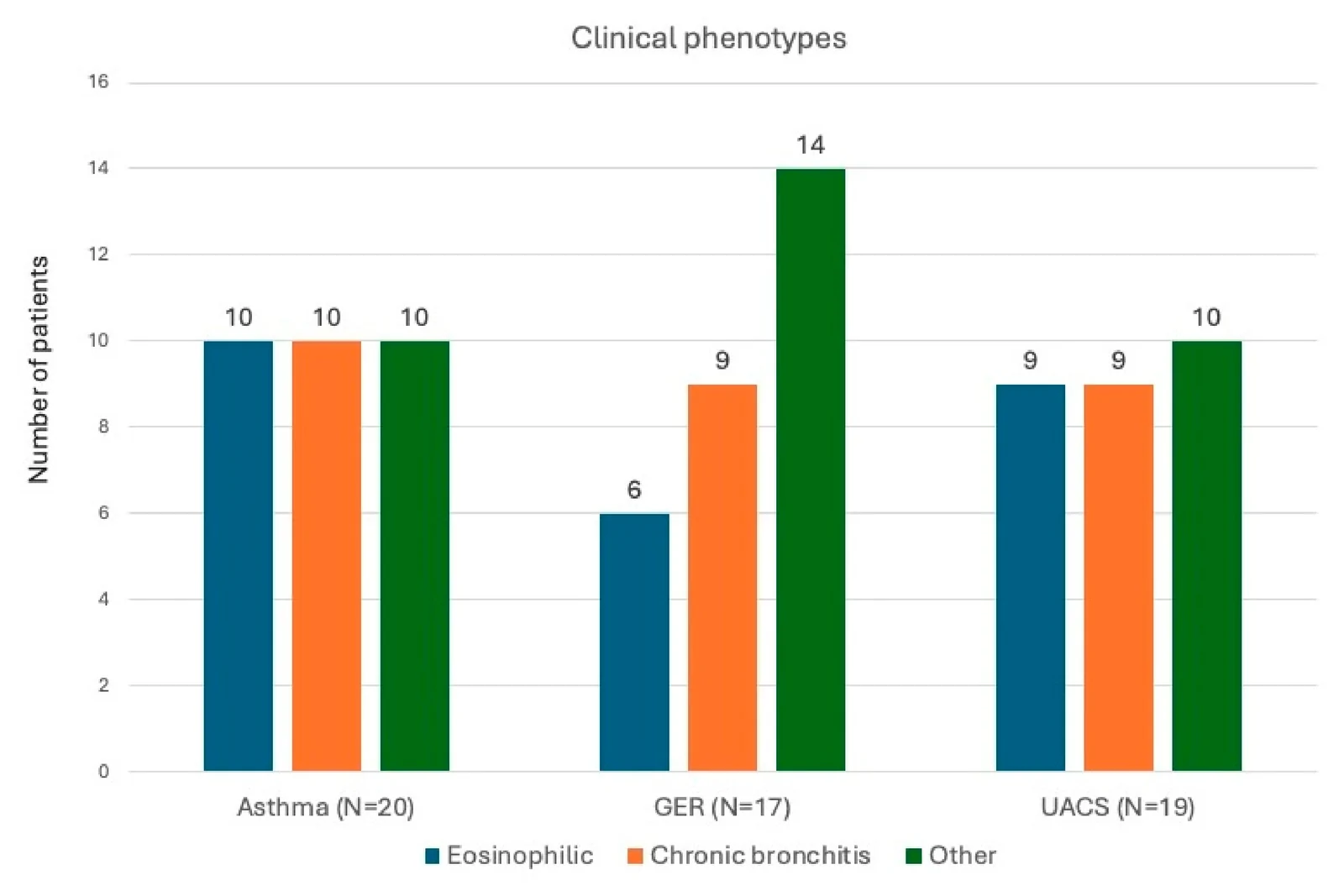
Distribution of clinical phenotypes in patients with asthma, GER and UACS. As clinical phenotypes and cough-related diseases might have coexisted, the number of identified phenotypes exceeded the number of patients.

Among patients with RCC and asthma, the distribution of clinical phenotypes was balanced. A similar pattern was observed in those with UACS. In contrast, among patients with RCC and GER, the clinical diagnosis of eosinophilic phenotype was less frequent, but these differences were not significant (*p* = 0.741) (Figure 4).

**Figure 5:**
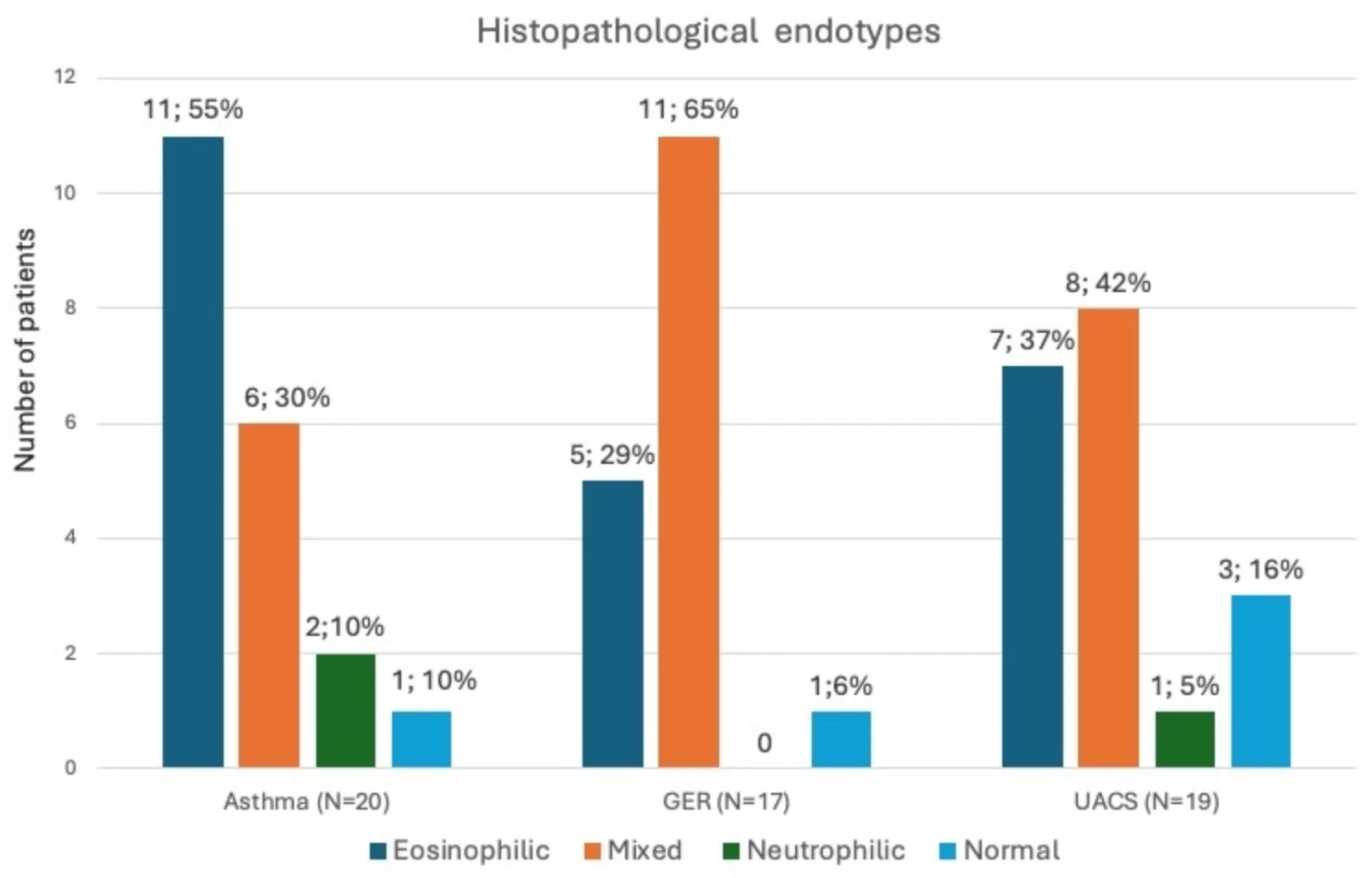
Distribution of histopathological endotypes in patients with asthma, GER and UACS. As cough-related diseases might have coexisted, the sum of the number of identified endotypes exceeded the number of patients.

The distribution of endotypes was more heterogeneous, regardless of the identified underlying cause or clinical phenotype, but these differences in the distribution of histopathological endotypes were insignificant (*p* = 0.386). Among patients with asthma, the eosinophilic endotype was the most common, whereas in patients with GER, the predominant pattern was the lymphocytic–neutrophilic endotype (Figure 5). In patients with UACS, both type 2 inflammation and mixed inflammatory endotypes were observed.

### 3.6. Assessment of Chest CT Abnormalities

High-resolution chest CT revealed slight abnormalities in 19 patients (61.3%), which identified airway inflammation: bronchial wall thickening (9 patients), areas of air trapping (9 patients), slight mucus plugs (8 patients), subtle peribronchial infiltrates (6 patients), and slight bronchiectasis (7 patients). In six patients, more than two types of abnormalities were present in HRCT. There was a moderate negative correlation between the total CT abnormality score and cough severity measured by VAS (rho= −0.45, *p* = 0.015). The prevalence of CT abnormalities was similar in patients with asthma, GER, or UACS. (Supplementary Table S2).

There were no differences in the prevalence of individual CT abnormalities between histopathological endotypes (Table 3).

## 4. Discussion

Our analysis highlights the complexity of clinical phenotypes in patients with RCC and shows that the association between clinical phenotype and the observed histopathological endotypes remains limited. This indicates that in this population, the clinical phenotype alone may be insufficient to predict the underlying histopathological pattern. These findings may influence both diagnostic strategies and the pathophysiological understanding of RCC. In our cohort, clinical phenotypes overlapped in one-third of patients, with the chronic bronchitis phenotype being the most common, followed by the eosinophilic phenotype. Subtle chest CT features suggestive of small-airway inflammation were frequent in patients with RCC but occurred independently of the histopathological pattern.

Although the literature frequently reports that patients with chronic cough exhibit features of airway inflammation and remodelling (such as subepithelial basement membrane thickening, goblet cell hyperplasia, and inflammatory cell infiltration [2,17]), our data suggest that, in RCC, there is no strict association between the eosinophilic or chronic bronchitis phenotype and the airway histopathological pattern. Moreover, these histopathological patterns in RCC were heterogeneous across patients with different treatable traits, including asthma, GERD or UACS. When interpreting these findings, the broad heterogeneity and overlap of clinical phenotypes across different RCC aetiologies should be taken into account.

As widely recognized, asthma is not a single disease entity but rather an umbrella term encompassing diverse clinico-inflammatory phenotypes [25]. In the context of chronic cough, cough-variant asthma (CVA) plays a key role, and its phenotypic heterogeneity is well documented. In the study by Rybka-Frączek et al., the paucigranulocytic phenotype was predominant, followed by eosinophilic and then neutrophilic patterns in patients with CVA [26]. Another study by Matsuoka et al. also documented heterogeneity of CVA phenotypes [27]; however, both studies were conducted in untreated CVA populations. In contrast, in our study, patients were treated according to the underlying cough aetiology, and all individuals with asthma received inhaled bronchodilators and corticosteroids.

In such a situation, demonstrating eosinophilic airway inflammation despite appropriate treatment may prompt an increase in the inhaled corticosteroid dose, the introduction of a short course of systemic corticosteroids, or consideration of biologic therapy. Conversely, the absence of eosinophilic inflammation in airway samples, together with the presence of remodelling features typical of RCC, suggests a potentially greater likelihood of cough reduction with therapies targeting RCC itself (non-pharmacological interventions, opiates, neuromodulators, gefapixant).

Based on our experience, another practical implication of identifying the histopathological endotype in patients with RCC may be the consideration of targeted antibiotic therapy in those with a neutrophilic or mixed inflammatory endotype. Recent studies have sought to characterize distinct phenotypes within RCC. Kang et al. successfully applied unsupervised machine learning techniques (K-prototype cluster analysis) to explore the profound heterogeneity of chronic cough based on demographic variables and COugh Assessment Test (COAT) questionnaire parameters. The authors identified four distinct clinical phenotypes with unique demographic and symptomatic profiles, including an isolated subtype of highly burdensome cough in older women [10].

Important insights into the complexity of chronic cough phenotypes also come from the longitudinal cohort study by Zhang et al., who identified potential treatable traits for six cough subclasses (including asthma, allergies, and active or passive smoking in the productive cough subgroup). The findings of this study emphasized the distinct nature of productive cough compared with dry cough, particularly regarding worse lung function trajectories [15,28].

In another study, the authors attempted to identify patient clusters using a newly developed TOPIC Questionnaire in individuals with chronic cough (47% with RCC), designed to assess cough-related sensations and triggers. They identified four distinct clusters based on reported sensory experiences and provoking factors: (1) a “high sensations burden” group, (2) a “vocal triggers” group, (3) an “eating triggers” group, and (4) a “need to throat clear” group [29].

All these studies illustrate ongoing efforts to identify distinct phenotypes among patients with chronic cough, including those with RCC, and they provide a valuable argument in our discussion. Together, they strongly support the notion that defining multidimensional, data-driven phenotypes is a crucial prerequisite for implementing individualized and effective therapeutic strategies in patients with RCC.

Although chest CT is not recommended as a routine diagnostic tool in patients with chronic cough [2], recent studies highlight its potential value in identifying certain treatable traits, such as hiatal hernia, bronchial wall thickening, or mucous plugs [30,31]. In this study, all patients underwent chest CT, and subtle small-airway abnormalities were present in most of them (61.3%). While our analysis did not reveal statistically significant differences in the prevalence of individual CT abnormalities across clinical phenotypes, this likely reflects the overlapping macroscopic sequelae of chronic airway inflammation. It may also be related to the relatively small sample size, since identifying CT abnormalities was not the primary aim of the study. Interestingly, we did not observe differences in the distribution of individual morphological features between the eosinophilic and chronic bronchitis histopathological patterns. On the contrary, Beck et al. demonstrated that mucus plugs were associated with type 2 airway inflammation in patients with chronic cough [18].

Our study has several limitations that need to be addressed. First, it was a single-centre study with a limited number of patients, without a control group. Second, all participants were receiving pharmacotherapy targeting the underlying aetiology of cough, and two-thirds of them were treated with inhaled bronchodilators and inhaled corticosteroids (ICS). This constitutes an important confounding factor, as ICS exert potent, pleiotropic immunosuppressive effects, and modify airway-remodelling processes [32]. Partial or complete resolution of cellular infiltrates under the influence of treatment may have substantially masked primary histopathological differences between distinct clinical phenotypes. Third, all participants had refractory chronic cough and were evaluated because bronchoscopy was clinically indicated, introducing a significant selection bias toward more complex and severe cases of RCC. Consequently, the study population may not be representative of the broader chronic cough population, Next, clinical phenotypes were defined using composite criteria that included biomarkers (FeNO, blood eosinophil count), symptoms, radiological findings, and coexisting diagnoses. Although these definitions reflect real-world clinical practice, some degree of phenotype misclassification cannot be excluded. It may partly explain the modest concordance observed between clinical phenotypes and histopathological endotypes Next, the cross-sectional design precludes conclusions regarding causality. It remains unclear whether the observed histopathological abnormalities contribute to cough persistence or represent consequences of long-standing cough and repeated mechanical airway injury. Finally, as this was a real-life study, histopathological assessment was performed by a single pathologist. Despite all limitations, we believe that the result of this study adds to the discussion on complexity character of inflammation and remodelling in patients with RCC.

## 5. Conclusions

In conclusion, our findings demonstrate that concordance between the clinical phenotype of refractory chronic cough and the underlying histopathological endotype is limited. Consequently, relying solely on clinical phenotyping may be insufficient to capture the cellular and structural heterogeneity of RCC. In selected patients, histopathological endotype analysis may provide additional insight into tissue-level pathobiology and help refine optimal therapeutic decision.

## Figures and Tables

**Table 1:** Baseline characteristics of patients with and without asthma.

Data	Patients with Asthma N = 20	Patients Without Asthma N = 10	*p* Value
Age (years)	54 (43.9–59.8)	56 (41.8–63)	0.792
Gender (F/M)	16/4	6/4	0.251
BMI (kg/m^2^)	26.2 (23.7–30.4)	29.4 (20.3–33.8)	0.961
Smoking history (NS/ExS)	13/7	6/4	0.354
Cough duration (months)	36.0 (17–120)	27 (12–72)	0.250
Character of cough (dry/productive)	12/8	7/3	0.165
Cough severity (VAS)	50 (17.4–83.6)	50 (38.9–68.9)	0.825
Quality of Life (LCQ)	10.6 (9.0–12.7)	11.25 (8.1–15.4)	0.567
Blood eosinophil count (cells/µL)	190 (98.5–276.6)	105 (64.8–15.9)	0.094
FeNO (ppb)	16 (10.3–24.6)	11.5 (8.1–15.14)	0.143

Values are presented as median and 95% confidential interval or number and percentages. BMI—body mass index, LCQ—Leicester Cough Questionnaire, VAS—Visual Analogue Scale, FeNO—fractional exhaled nitric oxide, F—female, M—male, NS—never smoker, ExS—ex-smoker.

**Table 2:** Concordance between clinical phenotypes and pathological endotypes in patients with RCC.

	Number of Patients	Number of Patients with Concordant Histopathological Phenotype	Accuracy
All patients	30	21	70%
Patients with eosinophilic phenotype	11	7	63.6%
Patients with chronic bronchitis phenotype	15	10	66.6%

**Table 3:** Distribution of abnormalities in chest CT in different histopathological endotypes.

Abnormality in Thoracic CT	Histopathological Endotype			
	Eosinophilic N = 10	Mixed Inflammatory N = 15	Other N = 5	*p* Value
Bronchial wall thickening	4 (40%)	7 (46.7%)	0	0.166
Bronchiectasis	4 (40%)	6 (40%)	0	0.223
Mucous plugs	3 (30%)	7 (46.7%)	0	0.153
Tree-in-bud opacities	3 (30%)	5 (30%)	0	0.330
Air trapping	2 (20%)	4 (26.7%)	2 (40%)	0.711
Total CT score (IQR)	1 (0–2)	1.5 (0–3)	0 (0–1)	0.331 *

Total CT score—sum of different abnormalities in chest CT in a single patient. * Analysis with Kruskal–Wallis test; for other analysis, Fisher’s exact test was used.

## Data Availability

The data presented in this study are available on reasonable request from the corresponding author due to privacy and ethical reasons.

## Supplementary Materials

The following supporting information can be downloaded at: https://www.mdpi.com/article/10.3390/jcm15176579/s1, Table S1: Results of differential cell count of bronchoalveolar lavage fluid; Table S2: Chest CT abnormalities in patients with different causes of refractory chronic cough. Figure S1: Diagnostic algorithm for common causes of chronic cough.
