# Supplementary material for: Bridging Clinical Phenotypes and Histopathological Endotypes in Refractory Chronic Cough
Source: J Clin Med. 2026 Aug 26;15(17):6579. doi: 10.3390/jcm15176579 (PMC13565798; doi:10.3390/jcm15176579)
Supplement: Supplementary file 1 [file jcm-15-06579-s001.zip › Clinical phenotypes vs histopahatol Supplementary tables 25.08.26.pdf]

Supplementary materials

Table S1. Results of differential cell count of bronchoalveolar lavage fluid.

Parameter	The whole group N=30	Patients with asthma N=20	Patients without asthma N=10	P value
Total cell count (cells x10^7/L)	3.6 (2.3-9.7)	4.5 (2.02 – 9.64)	2.7 (1.31 -12.33)	0.571
Macrophages %	74 (55.5-80)	75 (63.6 – 80.6)	62 (21.4 - 80.9)	0.471
Lymphocytes %	18 (12.8 -31.8)	15 (10.8 – 21.2)	23 (15.3 - 65.9)	0.116
Neutrophils %	4 (2.3-8)	4.5 (2.4 -6.0)	4 (1 - 12.3)	0.918
Eosinophils %	1 (0.3-4)	2 (0.4 – 4)	1 (0.1 – 2)	0.610

Data are given as median and IQR.

Table S2. Chest CT abnormalities in patients with different causes of refractory chronic cough

	Asthma N=20	GER N=16	UACS N=19	P value
Bronchial wall thickening	8 (40%)	4 (25%)	7 (36.8%)	0.621
Air trapping	6 (30%)	5 (31.25%)	6 (31.6%)	0.994
Mucus plugs	4 (20%)	6 (37.5%)	5 (26.3%)	0.500
Peribronchial infiltrates	4 (20%)	3 (18.7%)	3 (15.8%)	0.941
Bronchiectasis	3 (15%)	4 (25%)	3 (15.8%)	0.701

*Data are given as number of patients and percentages
